# Seroepidemiology of HTLV-1 and HTLV-2 Infection in Neyshabur City, North-Eastern Iran, during 2010-2014

**DOI:** 10.6091/.21.1.57

**Published:** 2017-01

**Authors:** Mohammad Salehi, Seyyed Khalil Shokouhi Mostafavi, Abdolmajid Ghasemian, Mahmoud Gholami, Abdolrahim Kazemi-Vardanjani, Mohammad Karim Rahimi

**Affiliations:** 1 Medical Diagnostic Laboratory of Neyshabour, Center of Medical, Pathological and Genetic Diagnostic Services, Iranian Academic Center for Education, Culture and Research (ACECR), Mashhad Branch, Mashhad, Iran; 2 Department of Microbiology, Islamic Azad University, Tehran Medical Science Branch, Tehran, Iran; 3 Department of Microbiology, Faculty of Medicine, AJA University of Medical Sciences, Tehran, Iran; 4 Department of Biology, Faculty of Sciences, University of Isfahan, Isfahan, Iran; 5 Shahrekord University of Medical Sciences, Shahrekord, Iran

**Keywords:** Human T-lymphotropic viruse, Seroepidemiology, Enzyme-linked immunosorbent assay, Iran

## Abstract

**Background:**

Retroviruses of human T-lymphotropic viruses (HTLV-1 and HTLV-2) have been demonstrated to be endemic in the north-eastern region of Iran. This study was aimed to determine the HTLV-1 and HTLV-2 prevalence among healthy individuals in Neyshabur City during 2010-2014.

**Methods:**

A total of 8054 blood samples were collected from healthy participants in Neyshabur, North-Eastern Iran. The blood samples were screened for the presence of specific antibodies against HTLV-1 and HTLV-2 by using ELISA according to the manufacturer’s instructions.

**Results:**

The overall seropositivity rate for HTLV-1 and HTLV-2 was found to be 6.55% (528 out of 8054) among participants.

**Conclusion:**

Both HTLV-1 and HTLV-2 were demonstrated to be at a high rate in healthy individuals. However, a smaller number of asymptomatic carriers were found in this study, as compared to those identified in previous investigations in the city.

## INTRODUCTION

Human T-lymphotropic viruses (HTLV-1 and HTLV-2), classified in the retroviridae family, are among the first identified species[1-3]. HTLV-1 and HTLV-2 are widespread all over the world and are endemic in different areas, including North-Eastern Iran[4-6]. According to a previous study, the rate of HTLV-1 infection has been reported to be less than 0.26% in Mashhad, North-Eastern Iran, while it does not exceed 0.34% in other areas of the country[7]. The prevalence of HTLV-1 infection in other countries such as Turkmenistan, Brazil, Spain, Korea and Japan was found to be 0.007%[8], 1.9%[9], 0.001%[10], 0.27%[11], and 0.12%[12], respectively. HTLV-1- and HTLV-2-infected carriers remain asymptomatic for a long time, serving as a potential source for the transmission of the disease[13]. The aim of this investigation was to determine the prevalence of HTLV-1 and HTLV-2 among healthy individuals in Neyshabur, North-Eastern Iran, during 2010-2014.

## MATERIALS AND METHODS

### Study population

A total of 8054 healthy individuals from Neyshabur, North-Eastern Iran, were included in this study. Serum samples (5 ml) were prepared from the individuals and stored at -20°C until the ELISA test.

### Serological assays and confirmation tests

Serum samples were screened for the presence of specific antibodies against HTLV-1 and HTLV-2 by ELISA (Dia.Pro Diagnostic Bioprobes, Italy) according to the manufacturer’s instructions[14].

### Statistical analysis

The SPSS software (version 20) was employed to analyze all data using chi-square and *t*-test. A *P<0.05* was considered to be statistically significant.

## RESULTS AND DISCUSSION

Of 8054 healthy individuals participated in the study, 1565 (19.4%) and 6489 (80.6%) were males and females, respectively. As shown in Table 1, the mean age of males and females was 46±3 and 51±3 years, respectively. The positivity of the samples was 6.55% (528 out of 8054), including 3.6% for HTLV-1 and 1.4% for HTLV-2. Table 2 indicates the total prevalence of HTLV-1 and HTLV- 2 in each year.

**Table 1:** Age- and sex-based distribution of individuals and overall HTLV-positive cases

Variable	No.	Positive cases (%)	Odd Ratio (OR)	OR (95%CI)	*P* value
Age (year)					
0-19	429	13(3.03)	Baseline		<0.0001
20-29	2556	49(1.92)	0.625	0.336-1.163	
30-39	2018	88(4.36)	1.459	0.807-2.637	
≥40	3051	377(12.36)	4.512	2.571-7.918	
Gender					
Male	1565	130(8.31)	1.386	1.128-1.704	0.002
Female	6489	398(6.13)			

**Table 2:** The annual prevalence of HTLV-1 and HTLV-2 investigated in this study

Year	Number	HTLV-1 (%)	HTLV-2 (%)	Total percentage
2014	Positive: 58	3.01	ND	3.01
	Total: 1350			
2013	Positive: 94	4.11	ND	4.11
	Total: 2337			
2012	Positive: 115	5.12	ND	5.12
	Total: 2188			
2011	Positive: 117	5.13	ND	5.12
	Total: 2057			
2010	Positive: 122	5.74	ND	5.74
	Total: 1789			

ND, not determined

Previous studies have revealed that HTLV-1 is endemic in North-Eastern Iran[15]. Another study in Neyshabur has indicated that the prevalence of HTLV-1 is 7.2% (35 out of 483)[16]. However, the rate of HTLV-1 seropositivity has gradually decreased from 1.97% in 1996 to 0.26% in 2014[17-19] in other regions of North-Eastern Iran. Similarly, the results of the present study demonstrated that the prevalence of HTLV-1 has decreased in Neyshabur from 2010 to 2014. In a survey carried out in Mashhad in 2012, the rate of HTLV-1 was detected to be 0.47%[20]. The seroprevalence of HTLV-1 did not exceed 0.19% in a study conducted by Safabakhsh *et al*.[7]. It seems that the reduction in HTLV-1 rate is mainly due to the improvement of blood donor selection and increased awareness among blood donors. However, in a study performed by Rafatpanah *et al*.[21] in Mashhad, it was revealed that the prevalence of HTLV-1 is 20% (10 positive samples), although no evidence of HTLV-2 infection was found among immuneblotted samples and nested PCR.

In the current study, over 3% of healthy individuals were positive for HTLV-1 in all five years. To the best of our knowledge, there is a small number of published data regarding HTLV-2 prevalence in Iran. Also, a lower rate of positive HTLV-1 infection was identified in the present investigation, when compared to a previously study in Neyshabur[22]. This finding highlights that Neyshabur is a major endemic region for HTLV-1. In addition, a higher prevalence of HTLV-1 was found in the age groups over 40 years, suggesting that there is a relationship between HTLVs and the age of individuals.

In the present study, a high rate of HTLV-1 among serum samples was detected using the ELISA test among healthy individuals in Neyshabur city during 2010-2014. The results from this study emphasize that HTLV is still an important endemic disease in Neyshabur. More importantly, the prevalence of HTLV-1 in Neyshabur was detected to be higher than other city (Mashhad) in all duration of this study, though being in a decreasing status compared to the previous reports.
